# Supplementary material for: Negative effects by mineral accretion technique on the heat resilience, growth and recruitment of corals
Source: PLoS One. 2024 Dec 30;19(12):e0315475. doi: 10.1371/journal.pone.0315475 (PMC11684729; doi:10.1371/journal.pone.0315475)

**S1 Fig. Map of the study area for the Mineral Accretion Technique (MAT) experiment.** Indicated are the position of the MAT (-4.650239, 39.387031) and Control table patches (-4.649854, 39.387534), relative to the power source and the nearby village Shimoni. Map data © OpenStreetMap contributors, available under the Open Database License (ODbL v1.0). Accessed from https://www.openstreetmap.org on 4 December 2024. Full ODbL available at https://opendatacommons.org/licenses/odbl/1-0/


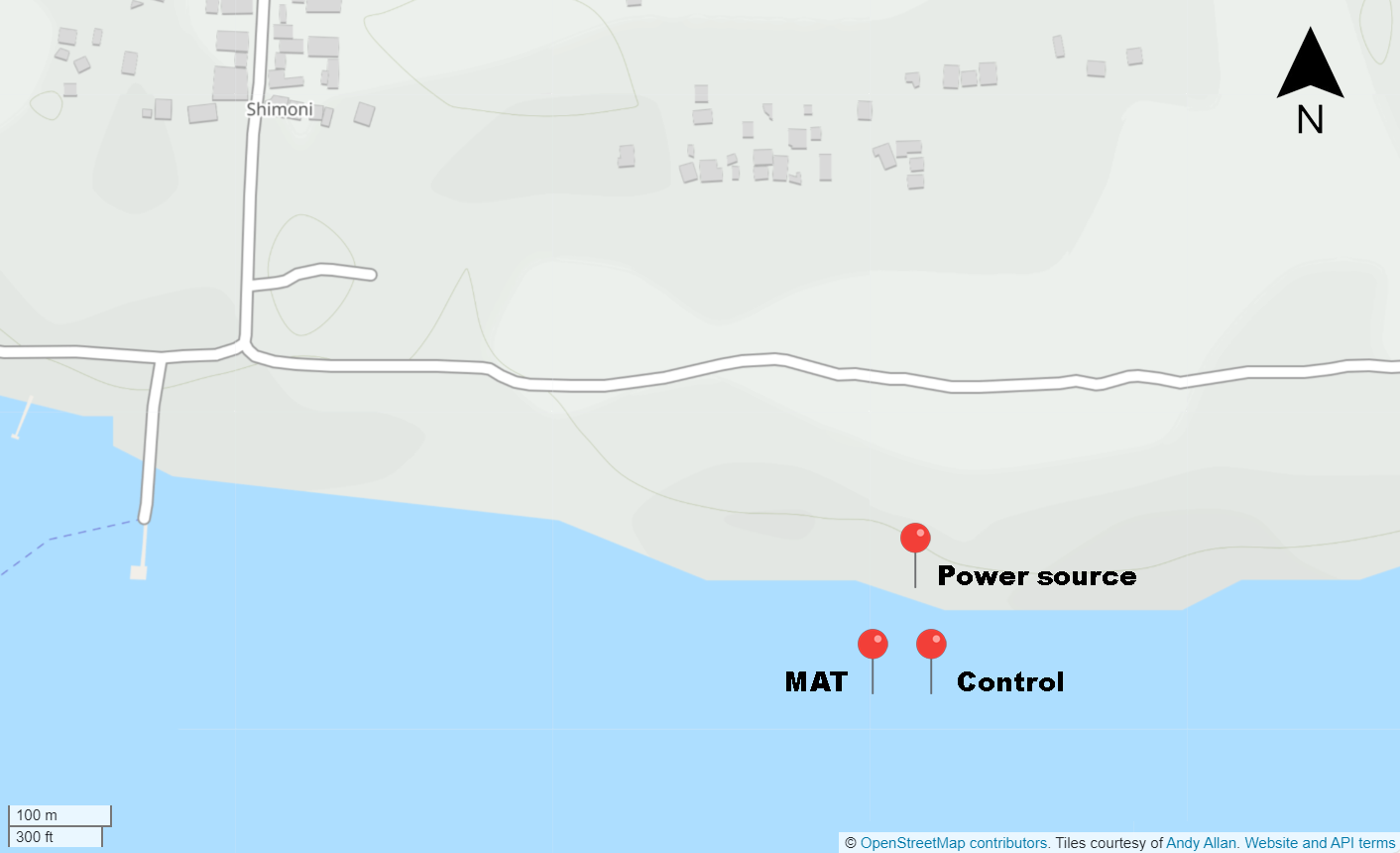

Supplement: S1 Fig — Indicated are the position of the MAT (-4.650239, 39.387031) and Control table patches (-4.649854, 39.387534), relative to the power source and the nearby village Shimoni. Imagery from Google Earth (Image © 2024 Airbus). (DOCX) [file pone.0315475.s002.docx]
