# Supplementary material for: Negative effects by mineral accretion technique on the heat resilience, growth and recruitment of corals
Source: PLoS One. 2024 Dec 30;19(12):e0315475. doi: 10.1371/journal.pone.0315475 (PMC11684729; doi:10.1371/journal.pone.0315475)

**S2 Fig. Pictures of the experimental setup. A)** A MAT (Mineral Accretion Technique) table with power cables running underneath. **B)** Top view of a Control table with the 16 fragments attached. **C)** Close-up of the main power cable splitting into 9 smaller cables, each running to a MAT table. **D)** Close-up of the midwater-suspended titanium anode. **E)** Close-up of a Pocillopora verrucosa fragment attached to a MAT table (a few days ago), showing the initial deposition of calcium carbonate. **F)** Close-up of a MAT table structure after several months, showing the thickening calcium carbonate layer.


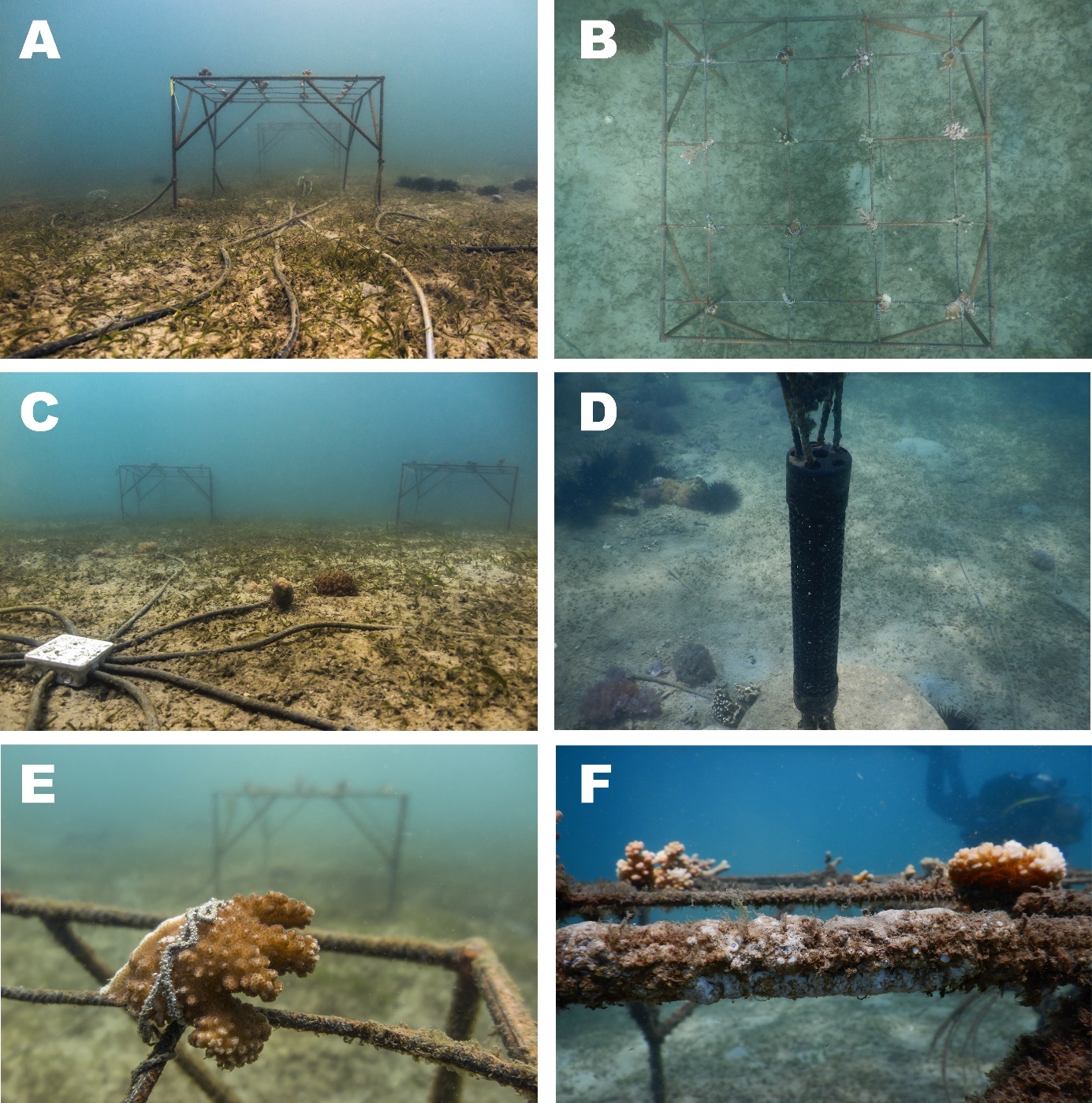

Supplement: S2 Fig — A: A MAT (Mineral Accretion Technique) table with power cables visible running underneath. B: Top view of a Control table with the 16 fragments attached. C: Close-up of the power cable splitting into 9 smaller cables running to each of the MAT tables. D: Close-up of the mid-water suspended titanium anode. E: Close-up of a Pocillopora fragment attached to a MAT table a few days ago, showing initial deposition of calcium carbonate. F: Close-up of table structure after 1 year showing the thick calcium carbonate layer deposited. (DOCX) [file pone.0315475.s003.docx]
