# Supplementary material for: Negative effects by mineral accretion technique on the heat resilience, growth and recruitment of corals
Source: PLoS One. 2024 Dec 30;19(12):e0315475. doi: 10.1371/journal.pone.0315475 (PMC11684729; doi:10.1371/journal.pone.0315475)
Supplement: S3 Fig — Both the Control and MAT tables were charged for the first month of the experiment, after which the Control tables were taken off the grid and moved 35 m away. The dashed line shows the initial circumference at the start of the study (for both Control and MAT tables). Converted to diameters (i.e. thickness), the initial diameter was 1.4 cm and, after 14 months, the diameter was 1.6 cm for the Control tables and 2.9 cm for MAT tables. (DOCX) [file pone.0315475.s004.docx]

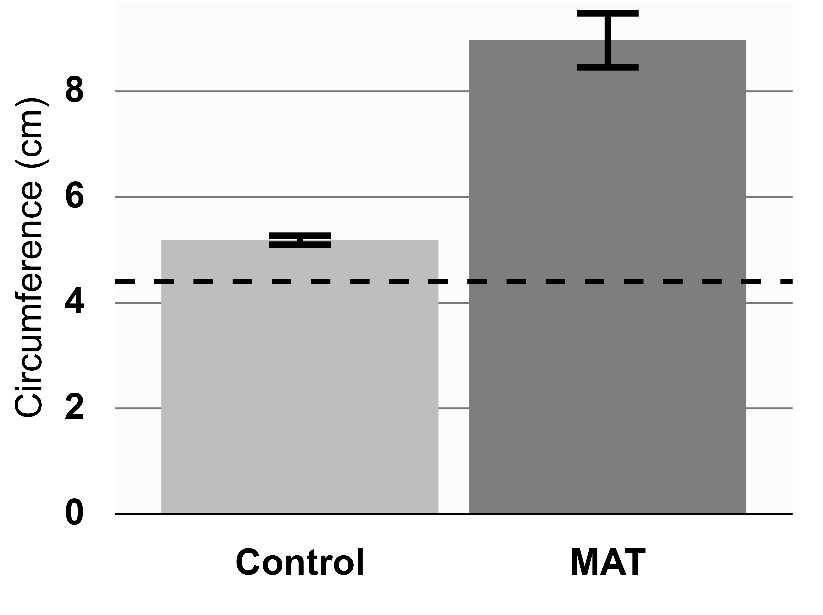


**S3 Fig. Mean (± SE) circumference of the metal rebar stakes that made up the tables of the Control and Mineral Accretion Technique (MAT) treatments after 14 months (n = 9).** Both the Control and MAT tables were charged for the first month of the experiment, after which the Control tables were taken off the grid and moved 35 m away. The dashed line shows the initial circumference at the start of the study (for both Control and MAT tables). Converted to diameters (i.e. thickness), the initial diameter was 1.4 cm and after 14 months the diameter was 1.6 cm for the Control tables and 2.9 cm for MAT tables.
