# Supplementary material for: Negative effects by mineral accretion technique on the heat resilience, growth and recruitment of corals
Source: PLoS One. 2024 Dec 30;19(12):e0315475. doi: 10.1371/journal.pone.0315475 (PMC11684729; doi:10.1371/journal.pone.0315475)
Supplement: S4 Fig — Error bars show SE (n = 9). A significant difference in start size was found between treatments using a linear model (χ2 = 7.992, df = 1, p = 0.00470). A Tukey post hoc test revealed that the start volume of Pocillopora verrucosa was significantly higher in the Control (p < 0.05, indicated by asterisk), whereas no significant differences were found for the other three species. (DOCX) [file pone.0315475.s005.docx]

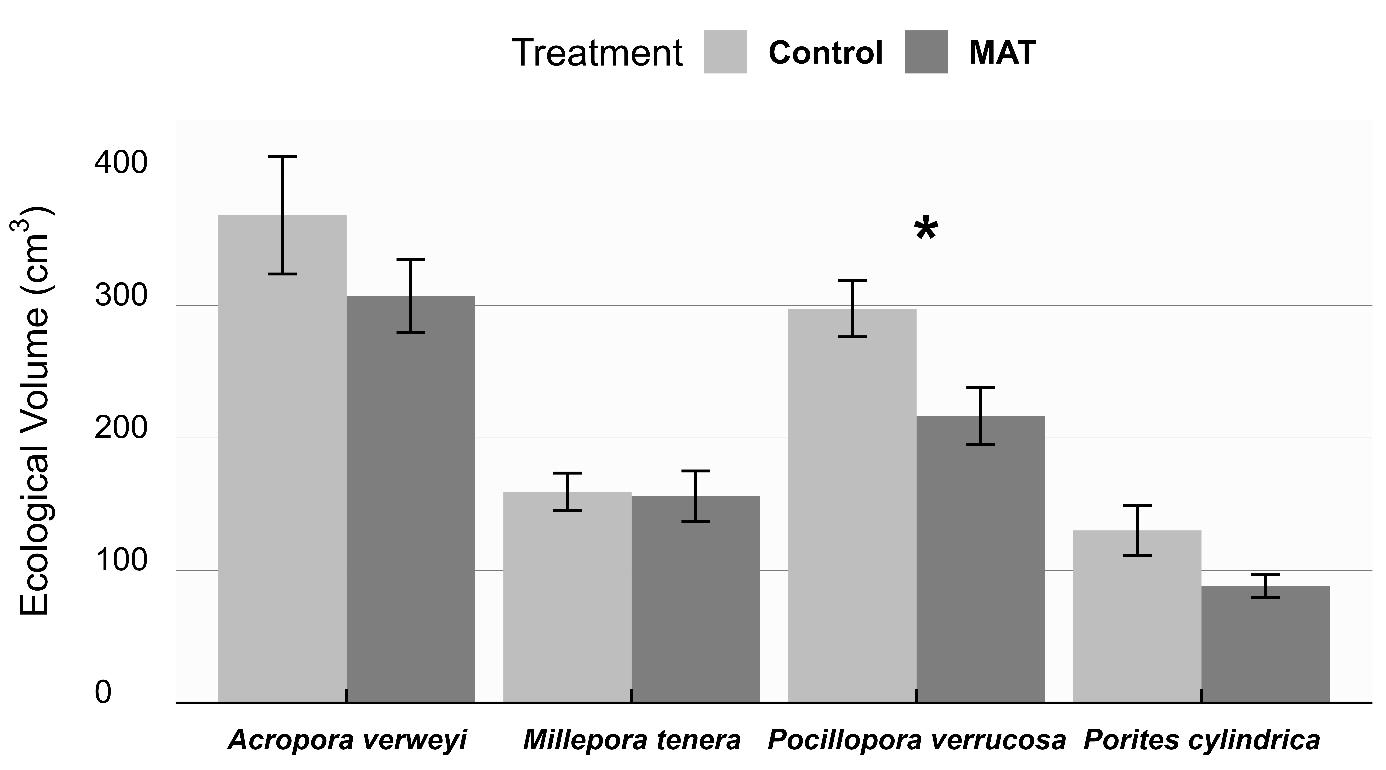


**S4 Fig. Average start sizes expressed as Ecological Volume (EV) of the four studied species, compared between Mineral Accretion Technique (MAT) and Control tables.** Error bars show SE (n = 9). A significant difference in start size was found between treatments using a linear model (χ2 = 7.992, df = 1, p = 0.00470). A Tukey post hoc test revealed that the start volume of Pocillopora verrucosa was significantly higher in the Control (p < 0.05, indicated by asterisk). No significant differences were found for the other three species.
