# Supplementary material for: Negative effects by mineral accretion technique on the heat resilience, growth and recruitment of corals
Source: PLoS One. 2024 Dec 30;19(12):e0315475. doi: 10.1371/journal.pone.0315475 (PMC11684729; doi:10.1371/journal.pone.0315475)
Supplement: S5 Fig — During this first month, the Control tables received the same amount of electricity as the MAT tables to form an anti-corrosion layer. No significant differences in live coral tissue were found between treatments for any species in this first month (X2 = 7.05, df = 3, p = 0.0702). (DOCX) [file pone.0315475.s006.docx]

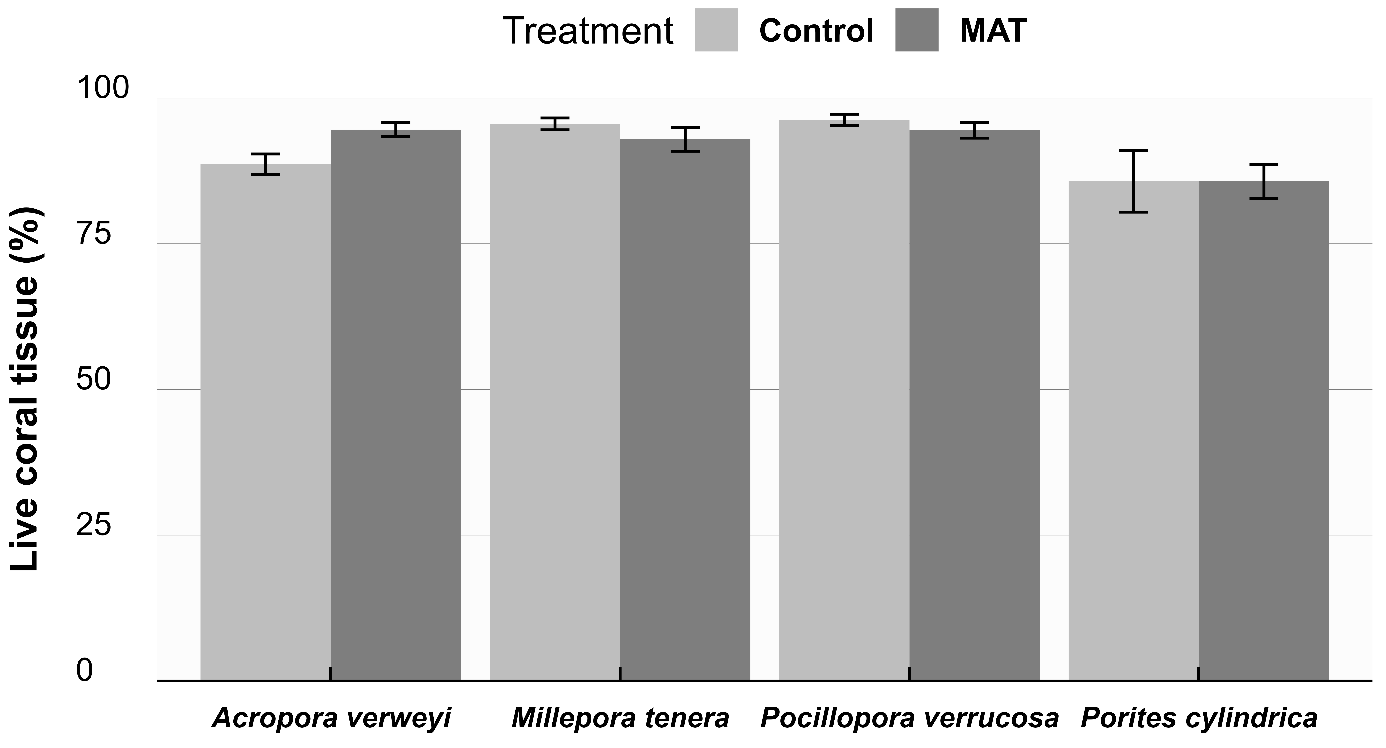


**S5 Fig. The percentage (mean ± SE) of live coral tissue of the four studied coral species after the first month (January 2020), compared between Mineral Accretion Technique (MAT) and Control treatment (n = 9).** During this first month, the Control tables received the same amount of electricity as the MAT tables to form an anti-corrosion layer. No significant differences in live coral tissue were found between treatments for any species in this first month (X^2^ = 7.05, df = 3, p = 0.0702).
