# Supplementary material for: Negative effects by mineral accretion technique on the heat resilience, growth and recruitment of corals
Source: PLoS One. 2024 Dec 30;19(12):e0315475. doi: 10.1371/journal.pone.0315475 (PMC11684729; doi:10.1371/journal.pone.0315475)
Supplement: S6 Fig — During this first month, the Control tables received the same amount of electricity as the MAT tables to form an anti-corrosion layer. No significant differences in growth rates were found between the treatments for any species in this first month (X2 = 1.86, df = 3, p = 0.601). (DOCX) [file pone.0315475.s007.docx]

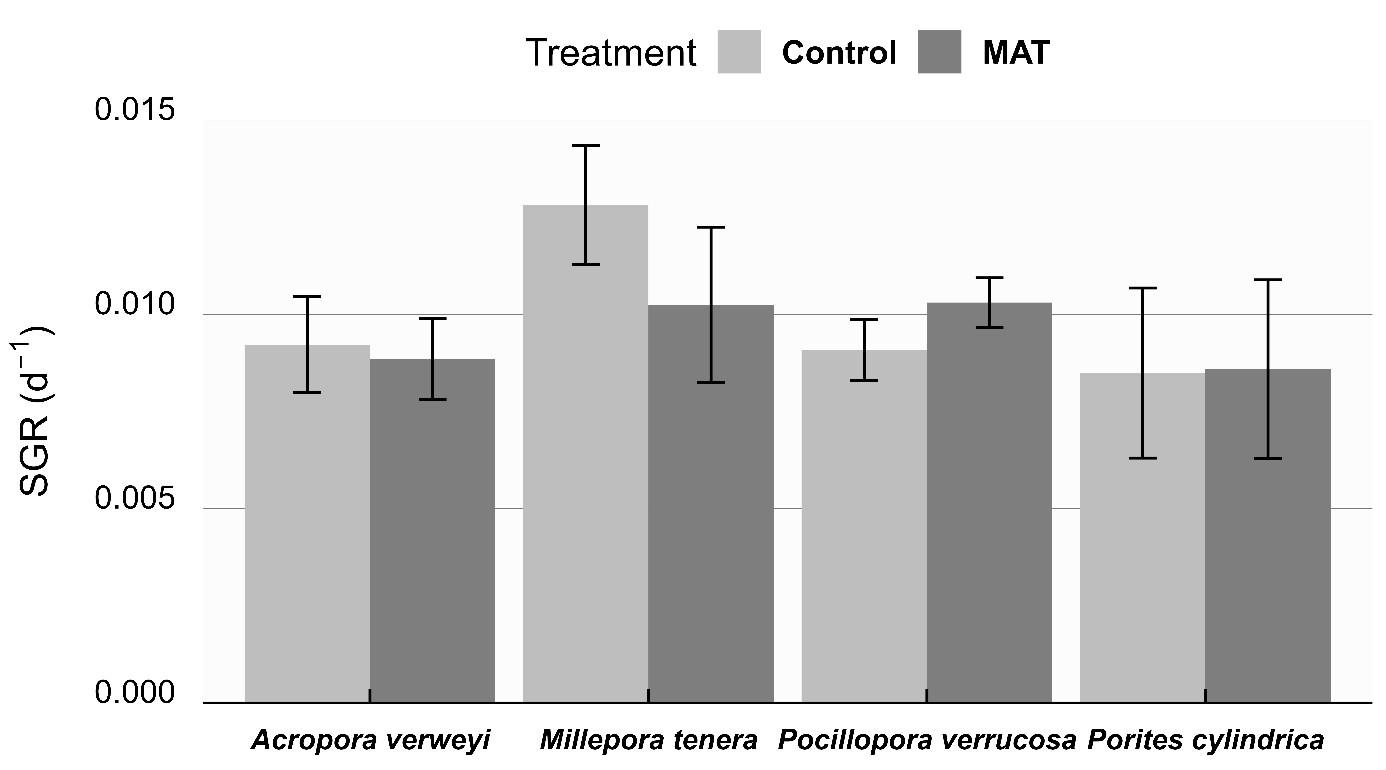


**S6 Fig. The mean (± SE) Specific Growth Rate (SGR) of the four studied coral species during the first month of the study (December 2019 – January 2020), compared between Mineral Accretion Technique (MAT) and Control structures (n = 9).** During this first month, the Control tables received the same amount of electricity as the MAT tables to form an anti-corrosion layer. No significant differences in growth rates were found between the treatments for any species in this first month (X^2^ = 1.86, df = 3, p = 0.601).
