# Supplementary material for: Negative effects by mineral accretion technique on the heat resilience, growth and recruitment of corals
Source: PLoS One. 2024 Dec 30;19(12):e0315475. doi: 10.1371/journal.pone.0315475 (PMC11684729; doi:10.1371/journal.pone.0315475)
Supplement: S7 Fig — For example, an EV increase of 200% means the fragment volume doubled, an EV increase of 100% means the fragment remained the same volume and an EV increase of 0% means a fragment lost all its volume (i.e. died). Note the logarithmic scale. A heatwave during the experiment killed all P. verrucosa fragments. Significant (p < 0.05) differences in EV increase between the MAT and Control treatment are indicated by an asterisk (*) for each species. (DOCX) [file pone.0315475.s008.docx]

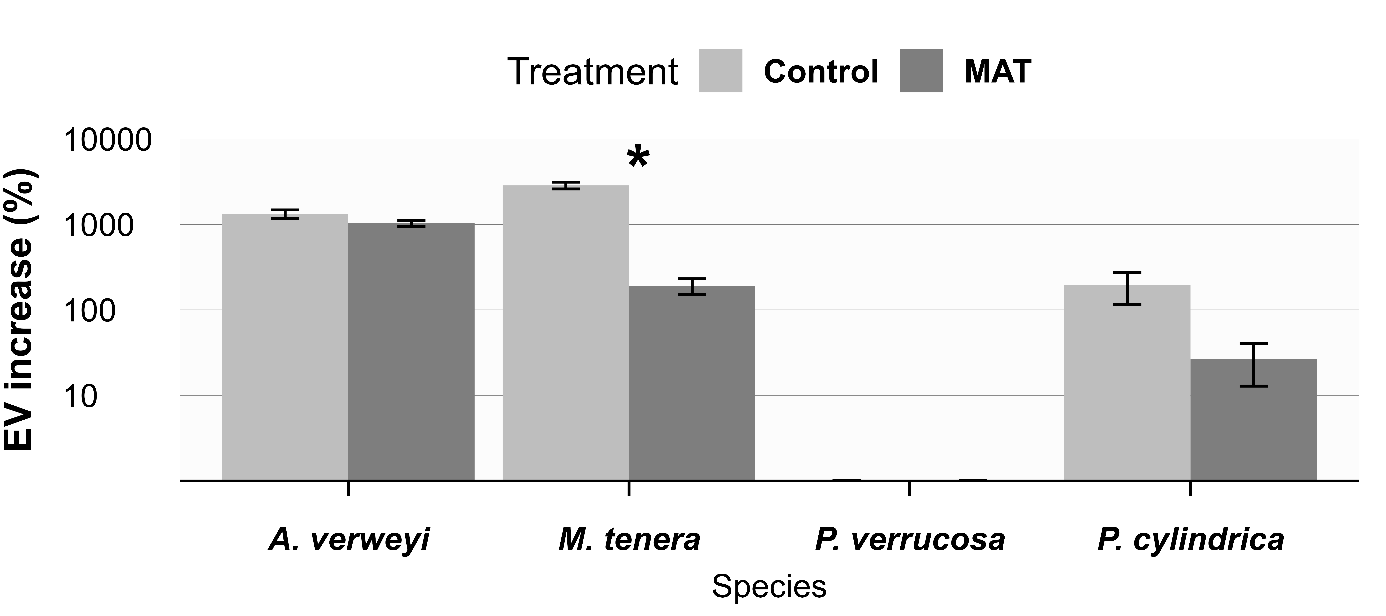


**S7 Fig. The mean (± SE) percentage increase in Ecological Volume (EV) of the four studied coral species (Acropora verweyi, Millepora tenera, Pocillopora verrucosa and Porites cylindrica) at the end of the 1-year experiment, compared between Control and Mineral Accretion Technique (MAT) treatment (n = 9).** For example, an EV increase of 200% means the fragment volume doubled, an EV increase of 100% means the fragment remained the same volume and an EV increase of 0% means a fragment lost all its volume (i.e. died). Note the logarithmic scale. A heatwave during the experiment killed all P. verrucosa fragments. Significant (p < 0.05) differences in EV increase between the MAT and Control treatment are indicated by an asterisk (*) for each species.
