# Supplementary material for: Negative effects by mineral accretion technique on the heat resilience, growth and recruitment of corals
Source: PLoS One. 2024 Dec 30;19(12):e0315475. doi: 10.1371/journal.pone.0315475 (PMC11684729; doi:10.1371/journal.pone.0315475)
Supplement: S8 Fig — Decreases in live coral tissue resulted in negative LER for some species. Significant differences in EV increase between the MAT and Control treatment are indicated by asterisks (*p < 0.05; ** p < 0.01; *** p < 0.001) for each species. (DOCX) [file pone.0315475.s009.docx]

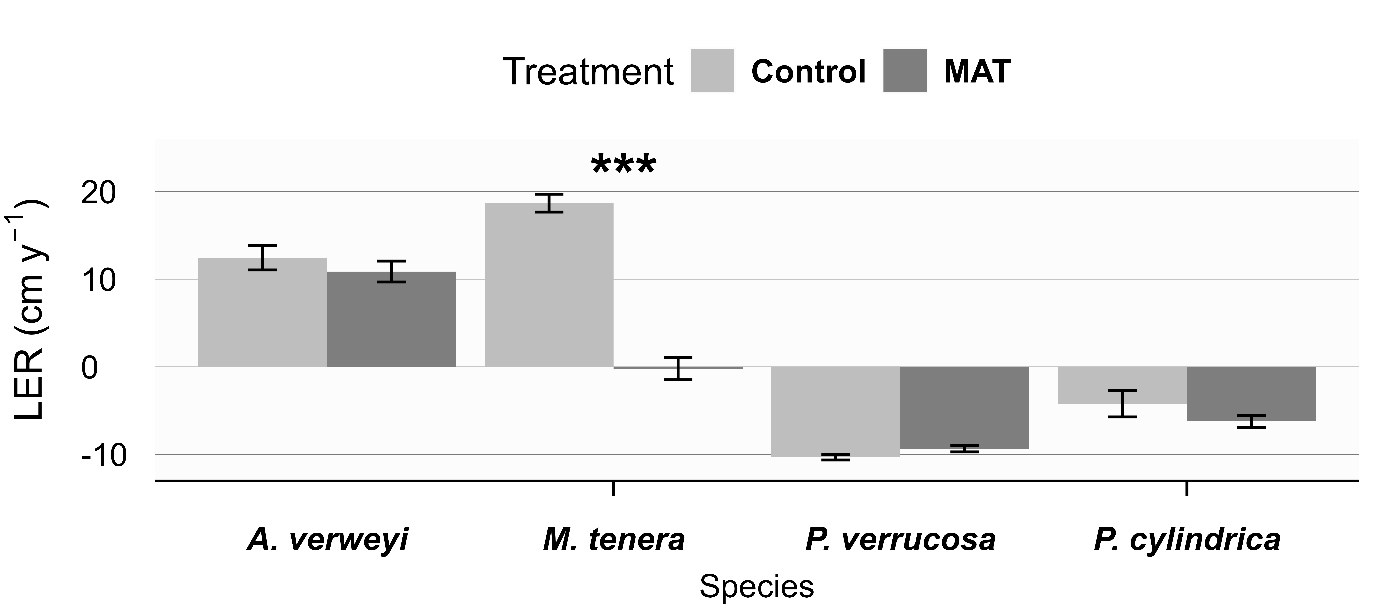


**S8 Fig. The mean (± SE) Linear Extension Rates (LER in cm y^-1^) throughout the study period for the four studied coral species (Acropora verweyi, Millepora tenera, Pocillopora verrucosa and Porites cylindrica), compared between the Control and Mineral Accretion Technique (MAT) treatment (n = 9).** Decreases in live coral tissue resulted in negative LER for some species. Significant differences in LER between the MAT and Control treatment are indicated by asterisks (*p < 0.05; ** p < 0.01; *** p < 0.001) for each species.
