# Supplementary material for: Negative effects by mineral accretion technique on the heat resilience, growth and recruitment of corals
Source: PLoS One. 2024 Dec 30;19(12):e0315475. doi: 10.1371/journal.pone.0315475 (PMC11684729; doi:10.1371/journal.pone.0315475)
Supplement: S9 Fig — The light blue line shows sea surface temperature as determined by NOAA from satellite data. The dark blue line shows the measured water temperature at the study location from March to September (using HOBO loggers). The dashed yellow line shows the long-term yearly mean maximum monthly temperature (MMM) plus 1 degree: this is used by NOAA as heat stress threshold. The dashed red line shows the accumulated heat stress as Degree Heating Weeks (DHW), following NOAA’s Coral Reef Watch (Liu et al., 2006). (DOCX) [file pone.0315475.s010.docx]

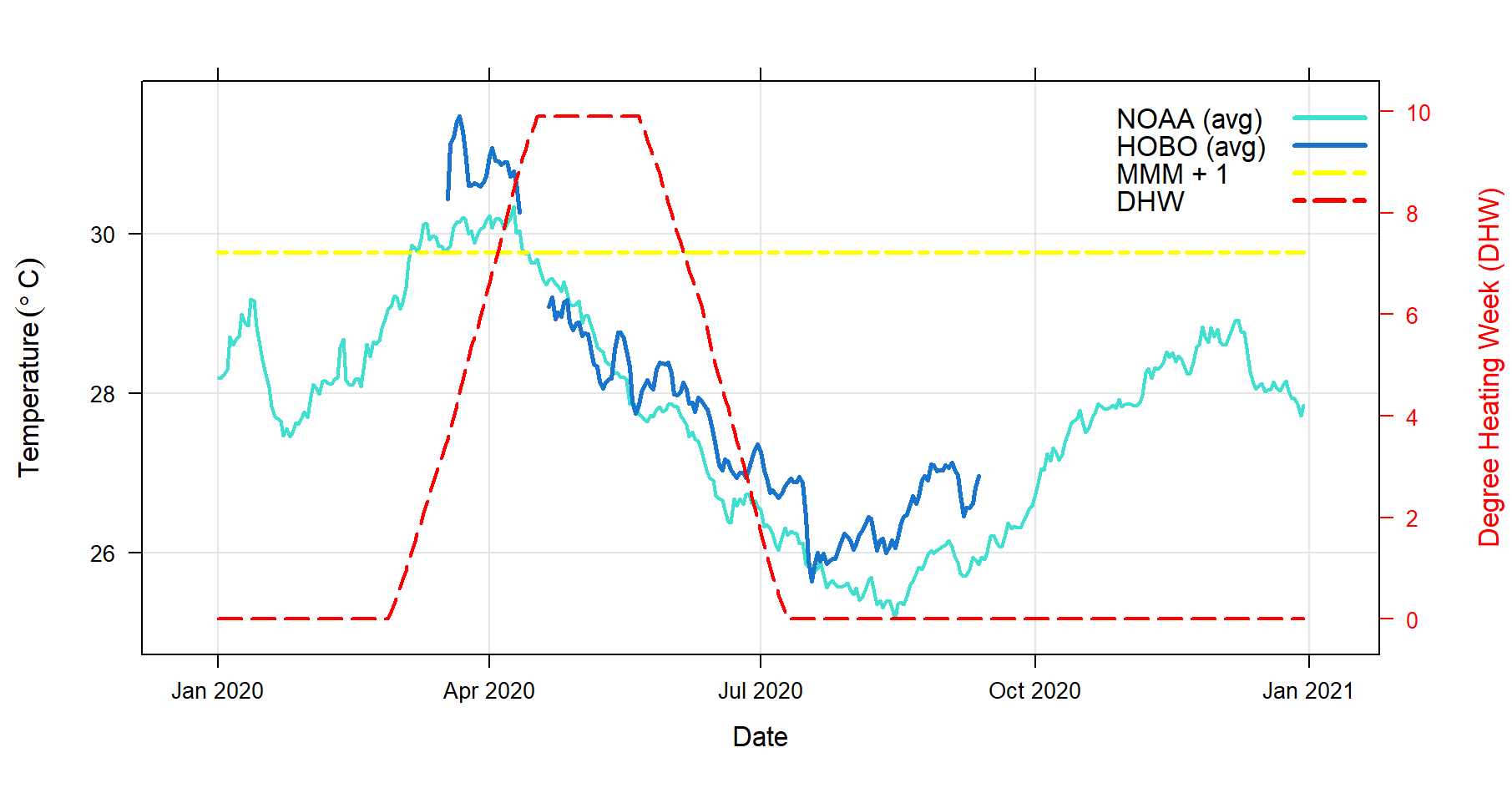


**S9 Fig. Overview of water temperature and heat stress during the one-year study. The light blue line shows sea surface temperature as determined by NOAA from satellite data.** The dark blue line shows the measured water temperature at the study location from March to September (using HOBO loggers). The dashed yellow line shows the long-term yearly mean maximum monthly temperature (MMM) plus 1 degree: this is used by NOAA as heat stress threshold. The dashed red line shows the accumulated heat stress as Degree Heating Weeks (DHW), following NOAA’s Coral Reef Watch (Liu et al., 2006).
