# Supplementary material for: Negative effects by mineral accretion technique on the heat resilience, growth and recruitment of corals
Source: PLoS One. 2024 Dec 30;19(12):e0315475. doi: 10.1371/journal.pone.0315475 (PMC11684729; doi:10.1371/journal.pone.0315475)
Supplement: S10 Fig — These pictures show growth in the month (June–July 2020) directly following a heatwave, when differences between MAT and Control were largest. Note the quick growth and encrusting of the coral on the Control table. Also note that the iron wire to attach the coral is overgrown on the Control table, but tissue around this wire died on MAT. (DOCX) [file pone.0315475.s011.docx]

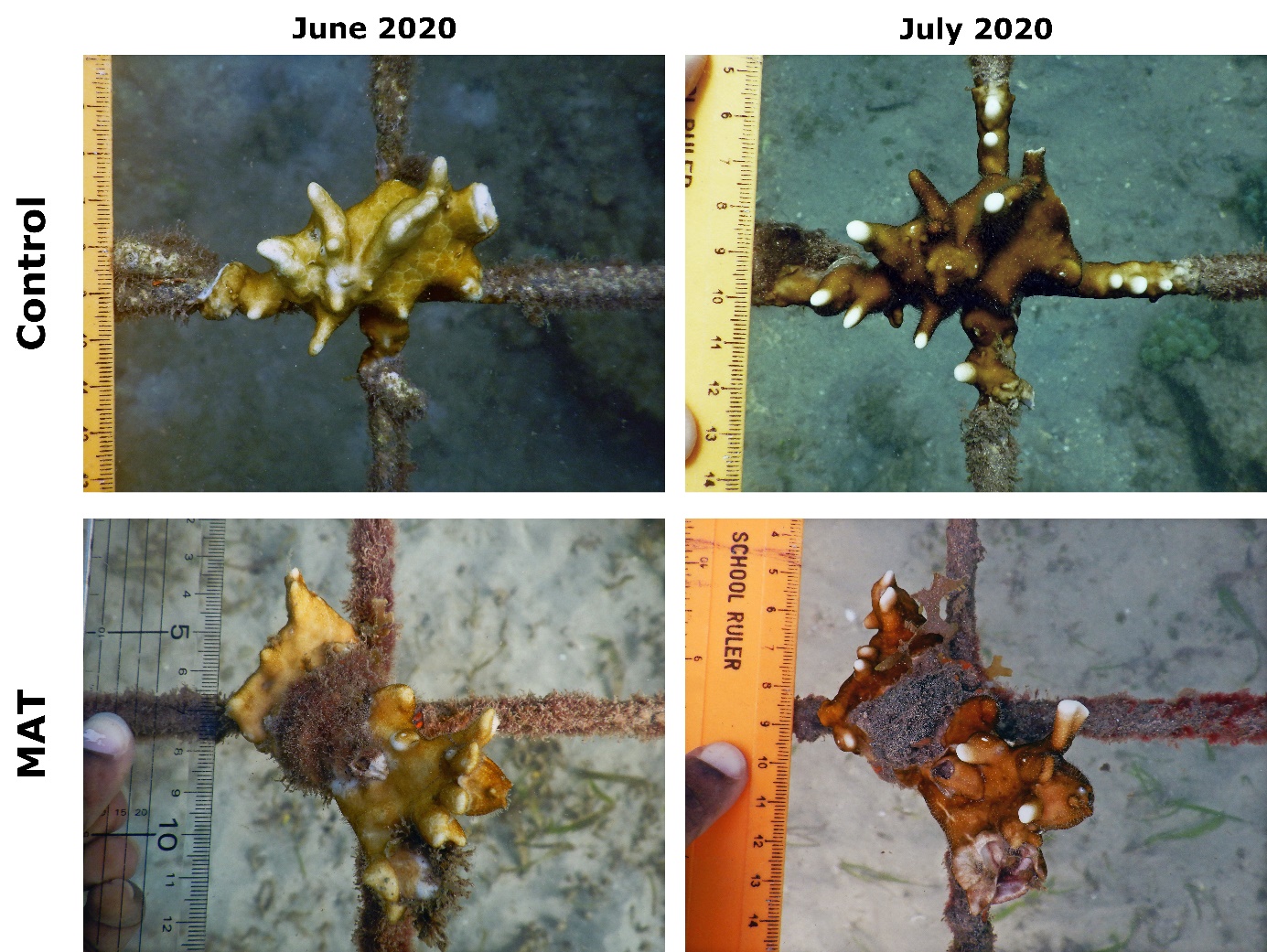


**S10 Fig. Representative photographs showing the differences in growth of the hydrozoan Millepora tenera on the Mineral Accretion Technique (MAT) and Control tables.** These pictures show growth in the month (June – July 2020) directly following a heatwave, when differences between MAT and Control were largest. Note the quick growth and encrusting of the coral on the Control table. Also note that the iron wire to attach the coral is overgrown on the Control table, but tissue around this wire died on MAT.
