# Supplementary material for: Negative effects by mineral accretion technique on the heat resilience, growth and recruitment of corals
Source: PLoS One. 2024 Dec 30;19(12):e0315475. doi: 10.1371/journal.pone.0315475 (PMC11684729; doi:10.1371/journal.pone.0315475)
Supplement: Attachment — Submitted filename: Response to Reviewers.docx [file pone.0315475.s012.docx]

Reviewer #1: Dear authors, dear editor,

The study “Lack of positive effects by mineral accretion technique on the bleaching resilience, growth and recruitment of corals” by Knoester and co-authors has investigated the effect of the Mineral Accretion Technique (MAT) on bleaching, live tissue cover, growth and recruitment of four coral species in Kenya over one year. They found more bleaching and lower growth rates of corals on MAT structures compared to control structures. This manuscript is very well written and describes the study and results very clearly (including nice visualisations of the results). The introduction includes all necessary background information about the MAT method. The discussion provides a very nice and concise comparison of the results with previous studies. My only major comment about this study is about the study design where the nine tables of the MAT setup were not true replicates as they have all been connected to the same power supply. This should be clarified in the text and also taken into account for the statistical analysis of the data. Apart from this, I only have some minor comments, especially in the methods sections where the authors should clarify a few things. The authors have provided all their data on GitHub, however I would recommend submitting the data to a data repository such as PANGAEA where the data files are checked according to the FAIR principle instead.

- **We thank Reviewer 1 for commending our research and the manuscript. Please find the responses regarding the issue of pseudoreplication and repository below.**

Abstract

The abstract provides a good summary of the study. However, due to the findings of the study, I would recommend re-rephrasing the title to “Negative effects of mineral accretion technique (MAT) on the bleaching resilience, growth and recruitment of three coral species” as the authors state that they didn’t just find no effect of MAT but even a significantly negative effect compared to the control.

- **Agree, we have changed the title as suggested (line 1).**

Introduction

The introduction is very well written and provides all necessary background information and controversial previous findings about this method for reef rehabilitation. It clearly states why this study is needed and the study aims as well as hypotheses.

- **Thank you for the positive feedback!**

Line 38: I would suggest to include some more information about the reasons why coral reefs have declined globally.

- **We added some more details here (line 37-38).**

Line 81: Change references to numbers for consistency.

- **Changed as suggested (line 80).**

Line 86: What do you mean by “condition”? Please clarify.

- **Meaning specified now in line 84-85.**

Lines 86-89: Please include information where this study took place.

- **Location info added in line 86.**

Methods

I think a map of the study site with the location of the MAT structures and controls would be helpful.

- **We have added a map to the supplementary information (S1 Fig) and referred to it in line 123.**

Line 100: I find it quite interesting that the pH varies so much at this site, can you give an explanation for this?

- **The cited study showed the lower pH values during the wet season at some specific locations, whereas pH measurements were more stable during dry season, so seasonality (river input?) appears to be a potential driver. In the study, the specific timing (hour of the day) of the pH measurements weren’t noted, so we can’t exclude further variation due to daily fluctuations, due to for example photosynthetic activity. No changes made to the manuscript.**

Lines 111-112: In my opinion, the nine tables of the MAT treatment cannot be viewed as true replicates if they were all connected to the same electricity source as indicated in Figure 1. Why was there not an individual electricity supply used for each table? Please clarify this in the text and also take it into account for the statistical analysis of the data.

- **The 100-m armoured cable running from the power source to the tables was by far the most expensive piece of equipment for this experiment, that is why a single cable was used and split at the end. The experiment costs would almost increase 10-fold if each table would get its own armoured cable to shore.**
- **To account for the potential non-independence between the nine tables connected to the same armoured cable, we added the power source as a hierarchical random factor, with the random factor of the table nested within it. This addition allows the new random factor, Power source, to address the non-independence between the nine tables connected to the same grid. The existing random factor, Table, continues to account for the non-independence between coral fragments on the same table. These changes were applied to the analyses of brightness (line 194-196) and condition data (line 206-208), resulting in only minor rounding adjustments to the model outputs, which do not affect our conclusions. However, due to a singularity in the model, it was not possible to apply these hierarchical random factors to the growth dataset. Therefore, the growth analysis remains unchanged, and we have noted this information in line 212-214.**
- **In addition, we stressed the issue of replication and power cables more elaborately in the discussion (line 341-345).**

Line 114: Please re-phrase as “was moved outside the electrical field of the MAT structure for the remainder of the study” to make it more clear, if I understood this correctly.

- **Understood correctly. Change applied as suggested (line 117).**

Lines 120-121: Unclear why the control tables were replaced and additional metal structures were connected to the grid, please specify.

- **The control tables were taken off the grid and moved away, to make sure no subsequent effects of electricity would influence the Control treatment. We believe this is clearly explained in line 115-120, so no further changes made to the manuscript on this (besides the adjusted line 117 as suggested above).**

Figure 1: Unclear what “5 Pod MAT” is, please clarify.

- **This explanation is in the figure description and main text, which we further clarified now (line 125 & Fig 1).**

In the timeline (C), it looks like coral brightness was only investigated during the marine heatwave, why was this not continued during the rest of the experiment to investigate coral recovery after the heatwave?

- **Due to logistical constrains during both the fieldwork and subsequent data quantification (both which are much more time intensive for the brightness data compared to the growth and condition data), we did not continue the brightness measurements for the full year. We believe any differences in heat resilience would be most evident during peak temperatures (which are well covered in our study). While the (rate of) recovery back to full colouration would also have been interesting, we believe the growth and condition data capture well how the corals were recovering after the heatwave. No changes made to the manuscript.**

The schematic of the experiment setup is very nice and helpful, however I think it would also be helpful for the readers to see actual photos of the structures/setup under water (or on land if underwater pictures are not available). Please provide some photos of the setup in the supplements if possible.

- **We added a series of underwater pictures on the experimental setup in the supplementary material (S2 Fig) and referred to this in line 123.**

Line 129: Unclear what the difference between the tables and pods are, please clarify.

- **As the pods are not of interest to this current study, we chose to not elaborate on the details of these structures. They were just added to prevent overcharging of the setup, as indicated in line 115-120.**

Line 142: Please define “short power outages”. How often and for how long did they occur?

- **Duration added in line 145-146: the outages never lasted longer than a few hours, a few times a month.**

Line 143-145: Are there any information about how MAT affects other organisms than corals as it might also be beneficial for e.g. growth of coralline algae, which might compete with corals for space?

- **In the original patent is it said to benefit all organisms, thus also fouling/competing organisms indeed. We haven’t found any studies or data on this though. As we removed all fouling from the structures in this study, this current publication will also not provide further clarity on this specific aspect, which is interesting for other studies to look into!**

Line 161: The nine tables cannot be treated as true replicates if they were all connected to the same power supply, please see comment above.

- **Please see elaborate answer above.**

Line 170: Why was the investigation of the brightness of coral fragments not continued after the heatwave/during the whole duration of the study?

- **Please see elaborate answer above.**

Line 178-181: How was the growth rate of the corals determined? I guess also by using the pictures and this is meant by “size measurements”? Please give some more details about the growth measurements.

- **We added some more basic info on the growth measurements, and more clearly linked this to the publication giving all details (line 181-184).**

Line 183: I would suggest to change it to “statistical analysis”.

- **Changed as suggested (line 189).**

Lines 187-189: Please also take into account that the nine tables cannot be treated as true replicates as they were all connected to the same power supply, therefore not only the 4 fragments on each table but also the 9 tables of each treatment are not independent. This also needs to be taken into account for the analysis of all other parameters. In addition, average values for each table should be used for the statistical analysis of all parameters and with table as random factor, not only for live tissue data to account for the independence of the 4 coral fragments on each table for all parameters.

- **Please see elaborate answer detailing on the same. Regarding taking the average: this will not be necessary if the Table is incorporated as random factor (as suggested), as this properly accounts for the non-independence of several fragments attached to the same table.**

Lines 204-205 and 208-211: I would move the description of growth measurements to the previous paragraph. Convert reference to number for consistency.

- **We moved the description and converted the reference as suggested (line 181-184).**

Line 210: I would suggest to also plot the linear extension rate data instead of presenting the data in a table.

- **We replaced the table by a figure as suggested (S8 Fig) and referred to it on line 216-217.**

Results

Lines 219-220: I would suggest to write “bleaching occurred in all four species with a peak in April” instead.

- **Wording changed as suggested (line 226).**

Figure 3: I would suggest to clarify the figure legend by not calling it “condition” but “live coral tissue cover”.

- **Legend is as suggested (Fig 3).**

Discussion

Lines 315-316 and 340: Change references to numbers for consistency.

- **Changed as suggested throughout manuscript (e.g. line 345).**

Line 314: I would suggest to delete “shockingly”.

- **Changed to ‘regrettably’ (line 319).**

Reviewer #2: Largely well written and a well-planned experiment. A few minor types and grammatical issues plus some more detail should be added to methods and a few considerations for discussion that I think are warranted prior to publication.

- **Thank you for the positive feedback.**

L100: remove ppt; Salinity is unitless. Also remove "between" before ranges with the en dashes throughout manuscript: salinity was 34-35m PH was 7.8-82, conductivity was...

- **We removed ppt as suggested (line 99). We stuck to our wording (‘varies between’) rather than replacing it with the suggestions of the reviewer (‘was’), as this more clearly indicates that these values are general values for the area, not measurements taken directly at the study site.**

L103: use abbreviation for meters after roman numerals

- **Changed as suggested, consistently throughout manuscript (e.g. line 101-102).**

L118: delete "between, and use date format of 17-19 December 2019; fix throughout manuscript

- **Changed as suggested, consistently throughout manuscript (e.g. line 121).**

L129: 235 V

- **Corrected (line 133).**

L148: using a comma before the last item in a series throughout would clarify complex series, such as this one. I would reorder to put the series in the series last for clarity.

- **Order changed for clarity (line 152-153).**

L172, which clause out of place; move to after brightness

- **Corrected as suggested (line 176).**

L195: the which clause should be a that clause without a comma: ..live coral tissue cover that may be explained by... (delete mainly)

- **Changed as suggested (line 202).**

L183-214: are there options you need to provide for any of these packages? If so, please provide the settings you used or make your code available so the analyses are repeatable by someone else

- **Yes the full code including all specific settings is available on Github (**[**https://github.com/ewoutknoester/MineralAccretionBleaching**](https://github.com/ewoutknoester/MineralAccretionBleaching)**) and will be included with the article as a Zenodo repository.**

L218-219: is something missing here: 10 degree heating weeks?

- **We added the information where this metric comes from (line 225).**

L227: this needs an antecedent; so they did not fully bleach on MAT? or other species did not or did frequently bleach? Please clarify this section

- **Section clarified (line 232-233).**

L235: however in the middle of sentences is not necessary and are a bit confusing as they seem to present contrary information but you are using them even for supporting information. I noted this issue elsewhere but did not comment. Please review your use of however throughout.

- **We changed the use of however where it was unnecessary or confusing (e.g. line 240 and line 16).**

L276: not a single benefit to coral performance that was evaluated in this study was found.... (unless you examined all possible benefits you should specify here so it is less likely you will be misquoted)

- **Agree, we changed this section to be more specific regarding our evaluated coral performance metrics (line 280-282).**

Results issues that need to be addressed and some thought for consideration. Given the timing of the warming/bleaching event, I think you need to have a few more caveats in there about the possibility of that event confounding the results for live coral tissue and growth rate. None of the differences between control and MAT were significant before the bleaching event. Therefore, the large differences you observed may not have been present if not for the apparent negative affect of MAT on bleaching, which may not be an effect MAT has on the coral but their zooxanthellae or other symbionts. I think other studies should look at whether the microbiome differs on control vs MAT. Does current have negative effects on single cells but lesser on multicellular organisms and thus the colonies?

- **We have stressed more thoroughly throughout the discussion that the negative effects of MAT manifested themselves only during the heatwave, while still showing that no positive effects were found before and after the heat stress (e.g. line 287, line 330). We have also incorporated the suggestion to look at the microbiome (line 353).**
